# Supplementary material for: Transfer Effects of a Multiple-Joint Isokinetic Eccentric Resistance Training Intervention to Nontraining-Specific Traditional Muscle Strength Measures
Source: Sports (Basel). 2023 Jan 4;11(1):9. doi: 10.3390/sports11010009 (PMC9865736; doi:10.3390/sports11010009)
Supplement: Supplementary file 1 [file sports-11-00009-s001.zip › sports-2041398-SI.pdf]

Supplement Table S1. Mean (SD), change scores, *p*-values, and Cohen's d effect size values for muscle function variables before (Pre) and after (Post) the training period for male participants.

Action	Variable	Pre	Post	Change score (%)	Cohen's d
Eccentron	Peak Force (N)	1791.91 (544.12)	2213.31 (484.56)	28.24	1.12
Leg Press	1 RM (N)	2491.00 (383.51)	2904.05 (424.89)	16.58	1.02
Knee Extensors (Biodex)	IsomPT (Nm)	190.80 (38.87)	202.58 (26.06)	6.17	0.36
	Ecc30 (Nm)	240.22 (44.67)	247.65 (37.66)	3.09	0.18
	Con150 (Nm)	157.74 (27.04)	158.37 (23.94)	0.40	0.02

Note: Cohen's d values compare within-group pretest and posttest differences and are identified as being small, moderate, and large on the basis of values of 0.2, 0.5, or 0.8 respectively. Ecc30 = Biodex knee extensor eccentric muscle action at 30°·sec^{-1}; Eccentron = maximal multiple-joint eccentric force on the Eccentron device; IsomPT = isometric PT of the knee extensors on the Biodex; Con150, Biodex knee extensor concentric muscle action at 150°·sec^{-1}.

Supplement Table S2. Mean (SD), change scores, *p*-values, and Cohen's d effect size values for muscle function variables before (Pre) and after (Post) the training period for female participants

Action	Variable	Pre	Post	Change score (%)	Cohen's d
Eccentron	Peak Force (N)	943.02 (332.37)	1496.09 (501.53)	58.65	1.33
Leg Press	1 RM (N)	1230.67 (276.60)	1534.64 (272.40)	24.70	1.11
Knee Extensors (Biodex)	IsomPT (Nm)	148.31 (36.26)	158.35 (25.26)	6.77	0.33
	Ecc30 (Nm)	189.14 (67.29)	203.22 (41.59)	7.45	0.26
	Con150 (Nm)	94.32 (22.48)	97.08 (17.00)	2.92	0.14

Note: Cohen's d values compare within-group pretest and posttest differences and are identified as being small, moderate, and large on the basis of values of 0.2, 0.5, or 0.8 respectively. Ecc30 = Biodex knee extensor eccentric muscle action at 30°·sec^{-1}; Eccentron = maximal multiple-joint eccentric force on the Eccentron device; IsomPT = isometric PT of the knee extensors on the Biodex; Con150, Biodex knee extensor concentric muscle action at 150°·sec^{-1}.
